# GelDrop Array High-Throughput Screening in Nematodes

**DOI:** 10.17912/micropub.biology.001809

**Published:** 2025-09-05

**Authors:** Xuan Wan, Paul W. Sternberg

**Affiliations:** 1 BBE, California Institute of Technology, Pasadena, California, United States

## Abstract

Nematode Growth Medium(NGM) plate–based screens consume substantial materials and time. We present GelDrop Array Screening (GelDrop), a gellan gum hydrogel droplet platform for *Caenorhabditis elegans* genetic crosses, mutation discovery, transgene integration, and small-molecule assays. Single animals are confined in discrete, bacteria ( OP50 )-supplemented droplets that sustain growth and reproduction for 2–3 days, providing screenable progeny while preventing escape and cross-contamination. Each 10-cm Petri dish supports 70–78 parallel screens. GelDrop can largely reduce agar use and labeling burden, boost throughput, and lower cost compared with conventional agar. The approach is simple, scalable, and compatible with standard microscopes and routine worm handling.

**Figure 1. GelDrop Array Screening:**
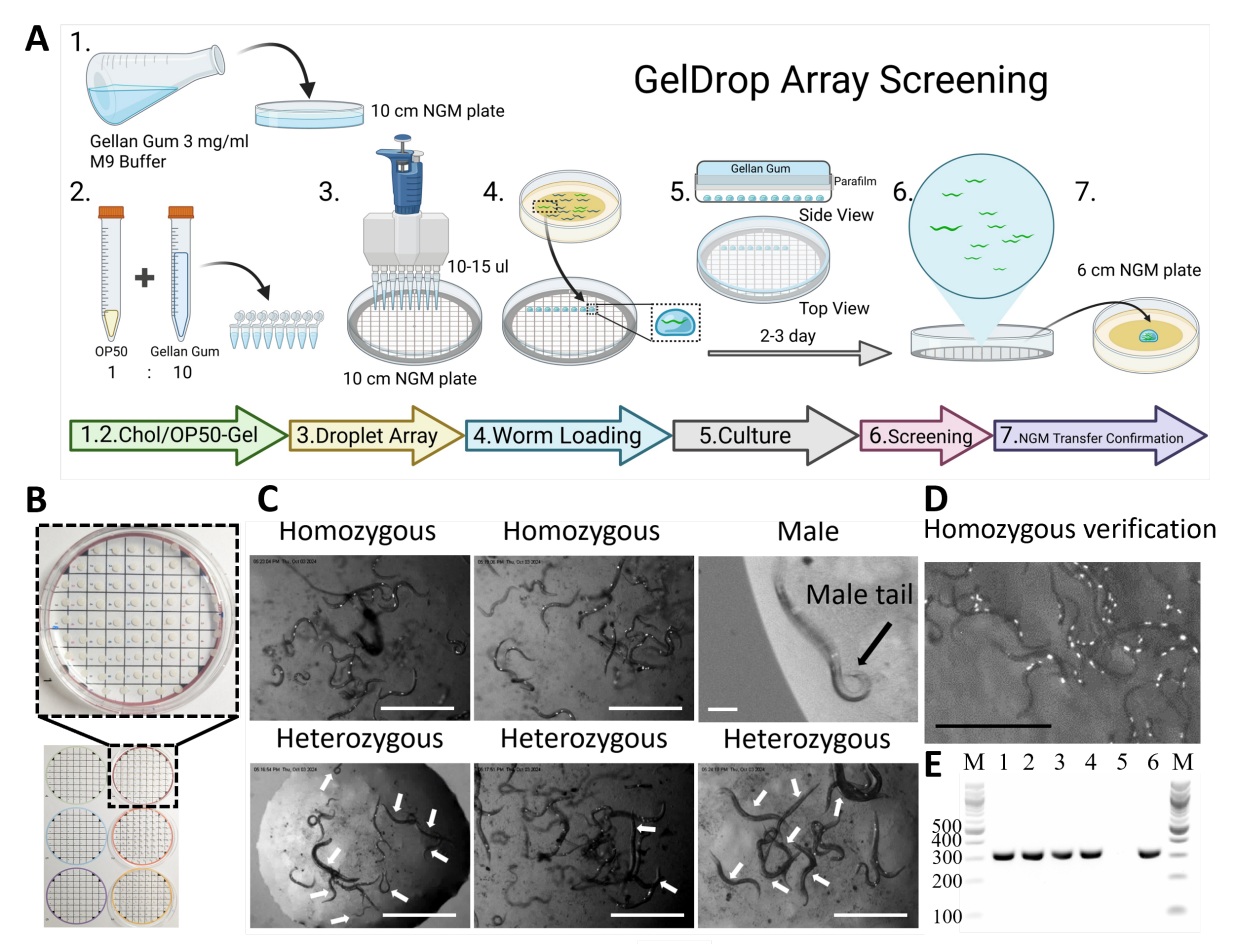
**(A)** Workflow schematic. Place a template grid beneath the lid to assign coordinates to each droplet. Prepare a 3 mg/mL gellan gum solution in M9 and cast a thin base layer in the bottom of a 10-cm dish. Mix the remaining gel with cholesterol and OP50 , then dispense 10-15 µL gellan gum feeding gel onto the inner surface of the lid with a multichannel pipette. One worm is loaded per droplet, cultured 2-3 days, screened on-plate, and positives are transferred by pipette to a standard NGM plate for confirmation/propagation. **(B)** A typical GelDrop plate showing a grid of ~70-78 spatially separated droplets with coordinate labels; examples of array templates are shown at the bottom. **(C)** Representative image demonstrating on-plate readouts: droplets scored as homozygous or heterozygous by a coelomocyte-expressed fluorescence protein marker. White arrows indicate animals lacking the coelomocyte fluorescence marker; only worms in the focal plane were annotated. Out-of-plane animals are unmarked. Male identification by tail morphology (black arrow). Top panels 1-2 and bottom panels 1-3 show strain PS8292 : *syIs563* [ *odr-10* p-GAL4(sk)-VP64, injection marker: coel::RFP] cross with N2 . Top panel 3 shows the CB4088 him-5 cross with N2 . F2 cross-progeny were singled and seeded one per droplet. Each image depicts a single GelDrop droplet. Scale bars are 100 µm for the male image and 1 mm for all other panels. **(D)** Abundant progeny on a standard NGM plate for confirmation that it is homozygous (Scale bars 1 mm). **(E)** PCR results for direct genotyping from GelDrop. Representative agarose gel showing target amplicons obtained after direct lysis of GelDrop worm mixtures with Proteinase K lysis buffer. Lanes: M, DNA ladder 1.5 µL; 1-4, N2 strain lysates prepared by mixing 2.0 µL, 1.5 µL, 1.0 µL, or 0.5 µL GelDrop worm mixture with 2.0 µL lysis buffer; 5, no-template control (NTC); 6, plate-grown control lysate; M, DNA ladder 3 µL. NEB 100 bp Ladder (#N3231; 100–1,517 bp; bright 500/1000 bp).

## Description

GelDrop Array Screening (GelDrop) is an arrayed hydrogel workflow that cultures single *Caenorhabditis elegans* in 10-15 µL gellan-gum droplets (with OP50 and cholesterol) arrayed on the plate lid, while a thin gellan gum layer is cast on the bottom of the dish to maintain humidity; the droplet array and base gel are separated and do not contact ( Figure 1A ). We adapted a gellan gum droplet array from liquid-culture protocols (Muschiol et al., 2007; Gilarte et al., 2015; Susoy & Samuel, 2023) that support normal growth, mating, navigation, and allows animals to swim freely (Susoy & Samuel, 2023; Wan et al., 2024). Culture format is known to shape physiology and behavior in *C. elegans* ; in particular, bulk liquid culture can yield animals with life-history and gene-expression states distinct from plate-grown worms (Çelen, Doh, & Sabanayagam, 2018; Hibshman, Webster, & Baugh, 2021), and males switch from parallel mating on plates to spiral mating in liquid (Susoy & Samuel, 2023). Accordingly, we use GelDrop as a practical, imaging-compatible assay and do not assume plate-equivalent physiology.

A 10-cm Petri dish reliably accommodates 70-78 spatially separated droplets arranged on a simple grid, enabling indexing of each animal by array coordinates in the template record ( Figure 1B ). After seeding one worm per droplet *, C. elegans* hermaphrodites feed, move freely, and produce progeny over 2-3 days; phenotypes can be scored directly in the droplet using a dissecting stereomicroscope ( Figure 1C ). Droplets of interest are aspirated and transferred intact to Nematode Growth Medium (NGM) plates for propagation or confirmation ( Figure 1D ) (Stiernagle, 2006). Surface tension keeps each animal within its droplet, preventing escape and cross-contamination. For the feeding mix, the gellan gum is allowed to solidify and cool down to room temperature, then gently fragmented and combined with OP50 and cholesterol; the resulting droplets remain cohesive and tolerate routine handling, including plate inversion and careful transport (Coutinho et al., 2010). Once the bacterial food is depleted, the droplet becomes clear, simplifying on-plate screening.

We also tested whether direct lysis of GelDrop samples can be used for PCR-based genotyping. To test whether GelDrop medium can be used directly for worm lysis and subsequent PCR, we combined GelDrop worm mixture with Proteinase K lysis and performed heat lysis followed by standard PCR. We evaluated four input volumes of the GelDrop mixture: 0.5 µL, 1.0 µL, 1.5 µL, and 2.0 µL. Each was mixed with 2.0 µL lysis buffer prior to heat treatment. Use 1.0-0.5 µL worm lysate as a template in a 20 µL PCR. Across all four conditions (corresponding to ~2.5-0.3% GelDrop by volume prior to PCR setup), PCR produced the expected target amplicons from both picked single/pooled worms (3-5 animals) and pipetted GelDrop suspensions, with no observable inhibition attributable to the gellan matrix. These results demonstrate that GelDrop is compatible with direct lysis and downstream PCR genotyping without additional cleanup steps and that a range of GelDrop-to-lysis ratios (0.5-2.0 µL GelDrop to 2.0 µL lysis) are suitable for routine use.

This GelDrop method supports rapid, high-throughput screening of large sample sets while minimizing plate handling. Because each assay uses microliter-scale droplets, GelDrop achieves high screening efficiency with minimal reagent use, making it cost-effective and environmentally sustainable. The array can be scaled with multichannel dispensing and adapted by altering droplet size, bacterial density, or by doping compounds directly into the droplet mix, enabling genetic crosses, mutation screening, transgene integration, RNAi screening (Ashrafi et al., 2003), and small-molecule or drug assays (O'Reilly, Luke, Perlmutter, Silverman, & Pak, 2014).

## Methods

**The following *C. elegans* strains were used:** PS8292 : *syIs563* ( *odr-10 p* -GAL4(sk)-VP64, injection marker: coel::RFP ) (Nava et al., 2023); N2 ; CB4088 : *him-5 ( e1490 )* . Animals were maintained on NGM plates seeded with OP50 at 20-22 °C.

**Base layer (for humidity control):** As shown in Figure 1A, prepare gellan gum (3 mg/mL) in M9. Dissolve by microwaving until it clears and fully dissolves. Pour 8 mL into a 10-cm dish (bottom) and allow it to set at room temperature. This thin gel maintains humidity and prevents droplet drying.

**Gellan gum feeding gel:** As shown in Figure 1A, from the same 0.3% gellan/M9, reserve ~50 mL for droplets and allow it to cool to ~35-40 °C (hand-warm). Add cholesterol to match NGM (50 µL of 5 mg/mL per 50 mL gel; final 5 µg/mL). Gently mix with concentrated OP50 ; 2 mL OP50 per 50 mL gel is a practical ratio and can be adjusted (1-3 mL per 50 mL) to meet feeding needs. Briefly break and mix them with a sterile stick, add cholesterol and OP50 , mix thoroughly, and keep at room temperature.

**Arraying droplets:** As shown in Figure 1A and B, place a template grid beneath the lid of the 10-cm Petri dish to assign coordinates to each droplet position. Using a multichannel or single-channel pipette, dispense 10-15 µL droplets of feeding gel onto the lid of the Petri dish based on the template grid. A 10-cm dish accommodates 70-78 droplets with ~5 mm spacing. Close the lid and wrap the plate with Parafilm to limit evaporation. Invert the dish so the droplet array faces up on the lid.

**Seeding and culture:** Transfer one worm into each droplet at the required developmental stage. Incubate at 20-22 °C for 2-3 days. As shown in Figure 1A and C, score growth, sex, genotype, drug responses, and fluorescence signal directly on the array using a dissecting stereomicroscope (fluorescence microscope optional).

**Recovery and confirmation:** As shown in Figure 1A and D, for any positive droplet, aspirate the entire droplet with a pipette and transfer it onto a standard NGM plate for confirmation and expansion.

**Genotyping sample collection and lysis protocol** : Dispense 2.0 µL of the Proteinase K lysis mix into each 0.2 mL PCR tube. Either pick 3-5 worms from the GelDrop array or pipette 0.5-2 µL of the GelDrop worm mixture directly into the lysis drop in the tube (tested input volumes: 0.5 µL, 1.0 µL, 1.5 µL, 2.0 µL; make sure at least 5-8 worms in the GelDrop worm mixture). Lysis program: 65 °C for 1 h, then 95 °C for 30 min to inactivate Proteinase K; hold at 4 °C. Use the resulting lysate directly as PCR template. Set up PCR reactions according to the manufacturer's instructions for polymerase. Add 1.0-0.5 µL of worm lysate to a 20 µL PCR reaction. Thermocycling conditions, primer sets, and reaction volumes should follow established genotyping protocols for the target locus. Primers used in this study: target a fragment of *gst-25* (5′→3′), 320bp: forward primer oHP877f, 5′-AATAGTTGCACCACCGCATTG-3′; reverse primer oHP878r, 5′-TGAATGCGACTTCCTGGTCC-3′.

**Notes.** Keep droplets at ≤15 µL to minimize merging of droplets and maintain plate sealing to prevent drying. The 0.3% gellan gum concentration yields firm, non-spreading droplets while allowing normal locomotion and feeding; modest adjustments (±0.1-0.2%) can be used to tune firmness if needed (Coutinho et al., 2010). Before screening, the plate can be briefly opened to let the droplets partially dry and firm up, which reduces worm movement and facilitates screening or imaging. As the droplets thin, worms settle at the bottom, making them easier to observe.

## Reagents

Gellan gum powder (Thermo Scientific (Fisher), Cat.J63423.30; Lot: W07J015); M9 buffer (3 g KH₂PO₄, 6 g Na₂HPO₄, 5 g NaCl, 1 mL 1 M MgSO₄, water to 1 L; autoclave) (Stiernagle, 2006); cholesterol stock at 5 mg/mL in ethanol (final 5 µg/mL); *E. coli* OP50 concentrated culture; 10- and 6- cm Petri dishes; multichannel pipette (optional); Parafilm. Lysis buffer (prepare and store as indicated): 50 mM KCl; 10 mM Tris-HCl (pH 8.3); 2.5 mM MgCl₂; 0.45% (v/v) Nonidet P-40; 0.45% (v/v) Tween-20; 0.01% (w/v) gelatin. Autoclave and store aliquots at −20 °C. Proteinase K (10 mg/mL). Immediately before use, prepare a working lysis mix by adding 1 µL Proteinase K (10 mg/mL) to 99 µL lysis buffer (final Proteinase K 0.1 mg/mL). Polymerase: Protocol for LongAmp™ Taq 2X Master Mix (New England Biolabs). DNA marker (M): NEB 100 bp DNA Ladder (#N3231; 12 fragments, 100–1,517 bp; brighter 500 & 1,000 bp bands).

## Data Availability

Description: Representative image demonstrating on-plate readouts: droplets scored as heterozygous by a coelomocyte-expressed fluorescence protein marker. . Resource Type: Audiovisual. DOI: https://doi.org/10.22002/wd2y1-w3r88 Description: Representative image demonstrating on-plate readouts: droplets scored as homozygous by a coelomocyte-expressed fluorescence protein marker. . Resource Type: Audiovisual. DOI: https://doi.org/10.22002/sjgvc-gn126
